# Noncontact Sleep Study by Multi-Modal Sensor Fusion

**DOI:** 10.3390/s17071685

**Published:** 2017-07-21

**Authors:** Ku-young Chung, Kwangsub Song, Kangsoo Shin, Jinho Sohn, Seok Hyun Cho, Joon-Hyuk Chang

**Affiliations:** 1 Department of Electronics and Computer Engineering, Hanyang University, Seoul 04763, Korea; kuyoungninezero@hanyang.ac.kr (K.C.); sentel103@naver.com (K.S.); 2 Intelligence Lab, LG Electronics Woomyon Research and Development Campus, Seoul 06763, Korea; ksoo.shin@lge.com (K.S.); jinho.sohn@lge.com (J.S.); 3 Department of Otorhinolaryngology-Head and Neck Surgery, College of Medicine, Hanyang University, Seoul 04763, Korea

**Keywords:** radar, vital signal, sleep stage, medical device, sensor fusion, microphone

## Abstract

Polysomnography (PSG) is considered as the gold standard for determining sleep stages, but due to the obtrusiveness of its sensor attachments, sleep stage classification algorithms using noninvasive sensors have been developed throughout the years. However, the previous studies have not yet been proven reliable. In addition, most of the products are designed for healthy customers rather than for patients with sleep disorder. We present a novel approach to classify sleep stages via low cost and noncontact multi-modal sensor fusion, which extracts sleep-related vital signals from radar signals and a sound-based context-awareness technique. This work is uniquely designed based on the PSG data of sleep disorder patients, which were received and certified by professionals at Hanyang University Hospital. The proposed algorithm further incorporates medical/statistical knowledge to determine personal-adjusted thresholds and devise post-processing. The efficiency of the proposed algorithm is highlighted by contrasting sleep stage classification performance between single sensor and sensor-fusion algorithms. To validate the possibility of commercializing this work, the classification results of this algorithm were compared with the commercialized sleep monitoring device, ResMed S+. The proposed algorithm was investigated with random patients following PSG examination, and results show a promising novel approach for determining sleep stages in a low cost and unobtrusive manner.

## 1. Introduction

High quality sleep is a profoundly important factor in an individual’s well-being because humans sleep nearly one third of their entire lives. Most people now live with a lack of sleep and tend to ignore the importance of sleep quality and sleep patterns, which can eventually lead to sleep disorders. Throughout the years, a number of devices has been launched to provide users with knowledge of their sleep hygiene [1,2].

One state-of-the-art technology for sleep quality assessment is polysomnography (PSG): a medical gold standard for evaluating sleep quality that takes place under the supervision of a trained professional sleep technologist. PSG is a long procedure that requires the use of various sensors and electrodes throughout the night [3,4]. Knowledge of sleep hygiene requires knowledge of sleep stages: awake, non-rapid eye movement (NREM) 1, NREM 2, NREM 3 and rapid eye movement (REM). PSG obtains knowledge on sleep stages from a long procedure in collaboration with sleep technologists and the PSG device. First, the PSG device extracts electroencephalography (EEG) information from EEG sensors attached to a patient’s head [5,6]. Then, the professional sleep technologist manually assesses results to compensate for errors the PSG device may make.

Despite the high reliability of PSG, its cost and obtrusiveness are inconvenient elements that prevent average users from using PSG as a long-term sleep analysis device. Hospital examinations are also expensive for users to invest in on a daily basis [7]. Another inconvenience of PSG is the obtrusiveness of its sensors and electrodes, which are attached throughout the patient’s body in a hospital bed. This environment prevents users from having their usual peaceful sleep, impedes correct measurement of sleep stages and diagnosis of sleep-related disorders. For these reasons, various commercialized products for sleep stage analysis have been launched on the market. Previously commercialized devices mainly used actigraphy [8], which analyzes body motion during sleep using accelerometers, electrocardiography (ECG) sensors [9], which measure the electrical activity of heart, and EEG sensors [10], which measure electrical activity in the brain. Nonetheless, such devices are still obtrusive.

To overcome these issues, Eliran Dafna et al. classified sleep stages using microphone sensors to detect breathing sounds and classify if a patient were awake or asleep, but sound data itself were not enough to determine NREM and REM sleep. The work in [11] used a single Doppler radar sensor to determine sleep stages (wake, NREM, REM) by extracting sleep-related vital signals, including breathing rate, heart rate and body motions [12,13,14]. However, the experiments were held in an environment in which the reference device and its manual were not certified by professional doctors or sleep technologists. Note that the reference device used in DoppleSleep is Hexoskin [1], which is not a certified product used as a gold standard for sleep stage classification. Furthermore, the subjects were not patients. The commercialized product ResMed S+ classifies sleep stages using multiple sensors and a complex algorithm that is not openly available. By combining the ideas of Tauhidur Rahman et al. [11], Eliran Dfana et al. [15] and ResMed [16], we suggest that the fusion of noncontact microphone sensor and radar sensor data-based advanced signal processing algorithms would likely enhance the accuracy of sleep stage classification. Moreover, most previously-proposed sleep stage classification methods are aimed at average users, not patients with sleep disorders who actually require sleep stage information. We propose the training of a random forest classifier algorithm based on extracted data from sleep disorder patients and statistical/medical knowledge.

In this paper, we propose a novel approach for sleep stage classification of sleep disorder patients supported by a low cost, noncontact, multi-modal sensor fused signal processing technique. The algorithm determines sleep stage by using sleep-related vital signals: breathing rate, breathing patterns, heart rate, body motions and snore sounds. Using a K-band 24-GHz Doppler radar transceiver as the main sensor, the system records phase differences in reflected electromagnetic waves from the limbs, torso and abdomen of the target to track breathing rate, heart rate and wake-related limb movement patterns. In a synchronous parallel fashion, the microphone sensor detects sound waves generated from the patient, which contains information on snore events and breathing patterns. After extracting selected features from multi-modal sensors, sleep stage classification is concluded via signal processing based on statistical and medical knowledge and uniquely-trained machine learning algorithms. A medical professional at Hanyang University, Seoul, South Korea, certified the reference device and manual script for the PSG data collected from sleep disorder patients.

The remainder of this paper is organized as follows. In Section 2, we meticulously describe the structure of the algorithm and introduce novel techniques for human adaptive sleep stage classification. In Section 3, we compare the sleep stage classification results and experiments with respect to a commercialized product, ResMed S+. Then, in Section 4, we discuss the results of the experiment and the limitations of this experiment. Finally, our conclusion is presented in Section 5.

## 2. Proposed Sleep Stage Analysis Algorithm

### 2.1. Pre-Processing of the Radar and Sound Signal

**Figure 1:**
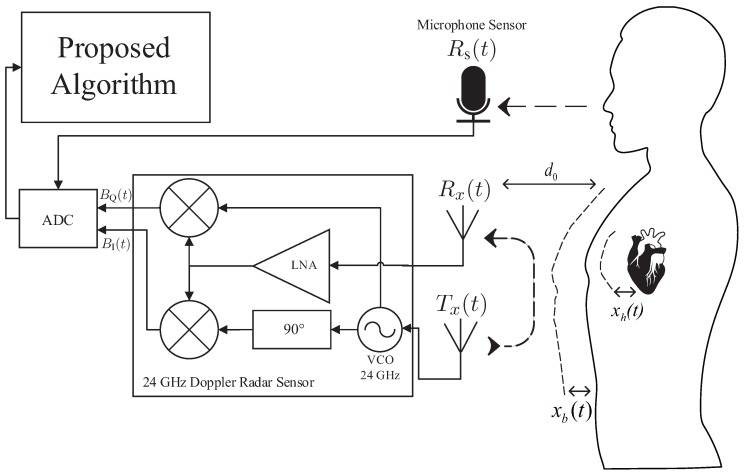
Block diagram of multi-modal sensors. LNA, low noise amplifier; VCO, voltage-controlled oscillator.

The proposed algorithm uses the RFBeam product K-LC5 transceiver sensor as the main sensor. K-LC5 is a low cost K-band 24-GHz continuous wave Doppler radar transceiver suitable for vital signal extractions [17]. Since the K-LC5 Doppler radar sensor extracts Doppler information based on the principles of the Doppler theory [18], phase changes in radiated or reflected electromagnetic waves can be detected from a moving object. Note that our target is a person, so the radar transceiver first transmits the transmitted signal, $T_{x}\left(t\right)$, towards the upper body of the patient, which is indicated as:

(1) $$T_{x}\left(t\right)=\cos\left(w_{0}t+\phi \left(t\right)\right)$$

where *t* represents time, $w_{0}$ is the 24-GHz voltage-controlled oscillator (VCO)-generated carrier frequency and ϕ(t) is the phase noise. The radar transceiver then obtains the received signal $R_{x}\left(t\right)$ from the target with a distance of $d_{0}$, which contains the following reflected Doppler information: breathing rate, heart rate and limb movements. The received signal is interpreted as:

(2) $$R_{x}\left(t\right)\approx \cos\left(w_{0}t-\frac{4\pi d_{0}}{\lambda_{T}}-\frac{4\pi x(t)}{\lambda_{T}}+\phi \left(t-\frac{2d_{0}}{c}\right)\right)$$

where λ${}_{T}$ is the wavelength of the transmitted signal $T_{x}\left(t\right)$, *c* is 3×$10^{8}$ m/s and x(t) is displacement of the target, which can also be viewed as $x\left(t\right)=x_{m}\left(t\right)+x_{b}\left(t\right)+x_{h}\left(t\right)$, where $x_{m}\left(t\right)$, $x_{b}\left(t\right)$ and $x_{h}\left(t\right)$ represent displacement of the limb, breathing and heartbeat, respectively. As displayed in Figure 1, after a received signal $R_{x}\left(t\right)$ passes through the low noise amplifier (LNA), it is converted to baseband after a mixing process multiplies the low noise amplified $R_{x}\left(t\right)$ with a carrier frequency generated by the equivalent VCO used for transmission.

The mixing process performs down conversion by disposing the carrier frequency 2πft down to an intermediate frequency (IF), which now contains only the desired data x(t). K-LC5 then uses a quadrature receiver to divide $R_{x}\left(t\right)$ into a pair of orthonormal baseband signals, in-phase and quadrature signals, by shifting the phase of 90${}^{\circ }$ before down conversion. The original $R_{x}\left(t\right)$ and phase shifted $R_{x}\left(t\right)$ are finally mixed with a VCO-generated carrier frequency. The output of the quadrature receivers, the in-phase signal *B*${}_{I}$(t) and quadrature signal *B*${}_{Q}$(t), are each interpreted as:

(3) $$B_{I}\left(t\right)=\cos\left(\theta +\frac{4\pi x(t)}{\lambda_{T}}+\Delta \phi \left(t\right)\right)$$

(4) $$B_{Q}\left(t\right)=\sin\left(\theta +\frac{4\pi x(t)}{\lambda_{T}}+\Delta \phi \left(t\right)\right)$$

where θ represents the phase difference that contains target distance information and Δϕ(t) is the phase noise. Since *B*${}_{I}$(t) and *B*${}_{Q}$(t) are 90${}^{\circ }$ phase shifted from each other, the null detection point problem is not an issue because either the in-phase or quadrature signal avoids the null point. To determine the optimal null-compensated signal among in-phase and quadrature signals, a channel selection process to determine the channel with a higher interquartile range is selected because a higher interquartile range implies further distance from the null-point [19].

Moreover, a data-driven noise reduction technique proposed by Erkelens et al. is used as the pre-processing method for the pre-processing of the sound signal, *R*${}_{s}$(t) [20]. The radar data are sampled to 1000 Hz because a previous study indicated that this sampling rate contains enough information to extract vital signals [21] compared to sound data that are sampled at 16,000 Hz [22]. Both sets of data are time-synchronized and have a frame size of 30 s, because PSG data from the hospital determines sleep stage every 30 s.

### 2.2. Step 1: Feature Extraction

**Figure 2:**
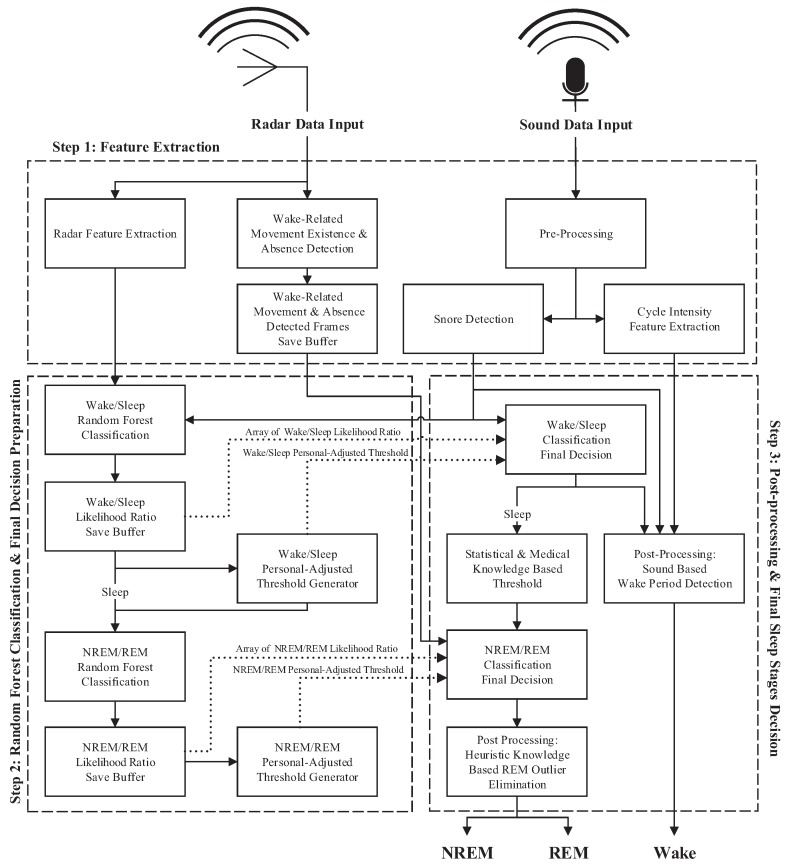
Block diagram of the proposed method.

As shown in Figure 2, the first step of the proposed algorithm is feature extraction of the received radar signals and sound signals from the pre-processing stage. Wake-related movement existence and absence detection (WRMEnAD) and radar feature extraction are performed from the channel-selected radar signal, whereas sound feature extraction is performed from pre-processed sound signals. During the radar feature extraction phase, the total number of the following 52 features is extracted.

#### 2.2.1. Forty Two Statistical Features of the Radar Signal

As proposed in [11], the radar signal passes through three customized bandpass filters that each extract movement, breathing and heartbeat signals. In addition, 14 statistical features (min, max, mean, root mean square, median, q1, q3, standard deviation, interquartile range, number of peaks, average distance between peaks, average amplitude of peaks, zero cross rate and linear regression slope) are extracted from each of the three signals.

#### 2.2.2. Two Spectral Features of the Radar Signal

Fourier transformation is applied to the breathing and heartbeat signals. The frequency bin located at the maximum amplitude in the frequency domain is determined as a spectral feature [4].

#### 2.2.3. Skewness and Kurtosis Features of the Radar Signal

Our newly-added features of skewness and kurtosis are each indicated as,

(5) $$F_{\mathrm{SKEW}}\left(i\right)=E\left[{\left(\frac{s_{i}\left(t\right)-\mu_{i}}{\sigma_{i}}\right)}^{3}\right]$$

(6) $$F_{\mathrm{KURT}}\left(i\right)=\frac{E[{\left(s_{i}\left(t\right)-\mu_{i}\right)}^{4}]}{E{\left[{\left(s_{i}\left(t\right)-\mu_{i}\right)}^{2}\right]}^{2}}$$

where error represents expectation and $\mu_{i}$ and $\sigma_{i}$ are defined as the mean and standard deviation of the *i*-th frame of the radar signal $s_{i}\left(t\right)$, respectively.

#### 2.2.4. Three Singular Value Decomposition Features of the Radar Signal

The singular value decomposition (SVD) features are singular values, $\sigma_{SVD1}$, $\sigma_{SVD2}$ and $\sigma_{SVD3}$, which are derived from the following equation:

(7) $$A=U\Sigma V^{T}$$

where *A* is the radar feature matrix, *U* is a unitary matrix of left singular vectors of *A*, Σ is a rectangular matrix of singular value matrix of *A* and $V^{T}$ is a transposed unitary matrix of right singular vectors of *A*.

#### 2.2.5. Three Principal Component Analysis Features of the Radar Signal

The principal component analysis (PCA) features $w_{1}$, $w_{2}$ and $w_{3}$ are the three column vector features achieved from a centralized covariance matrix of radar features Cov(A) calculated as:

(8) $$\mathrm{Cov}\left(A\right)=\frac{{\left(A-\mu_{A}\right)}^{T}\left(A-\mu_{A}\right)}{\left(N-1\right)}$$

where *N* indicates the number of rows.

#### 2.2.6. Wake Related Movement Existence and Absence Detection Flag

**Figure 3:**
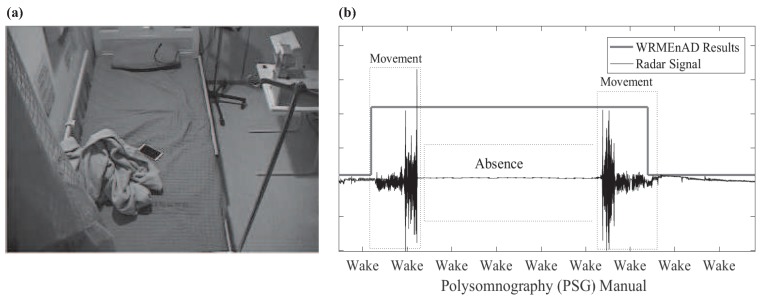
The wake-related movement existence and absence detection (WRMEnAD) algorithm: (**a**) absence of the patient during sleep; (**b**) performance of the WRMEnAD algorithm.

In synchrony with the extraction procedure, the proposed WRMEnAD algorithm detects wake-related motions with a high probability of indicating that the person is awake. For example, these include tossing and turning of a patient during sleep [23]. The WRMEnAD algorithm is also capable of detecting the absence of a patient within the sleep environment. The idea behind this algorithm originated from Figure 3a, from which we realized that some patients leave their bed during sleep because of hygiene issues or sleep disorders, such as somnambulism [24]. Wake-related movement is detectable by extracting the energy-based level-cross-rate feature from the radar signal, which is then compared to the heuristic threshold, $\gamma_{\mathrm{MOVE}}$. If the algorithm detects wake-related movements in a certain frame, it is labeled as one; otherwise, it is labeled as zero. Likewise, the detection of absence can be achieved by analyzing the energy of the *i*-th frame of the radar signal E(*i*) with respect to the heuristic absence threshold $\gamma_{\mathrm{ABS}}$. If a frame is determined as an absence, it is also labeled as one since the gold reference PSG determines absence as awake (Figure 3b).

#### 2.2.7. Sound Features

During the sound feature extraction phase, a data-driven noise reduction technique proposed by Erkelens et al. [20] is used as the pre-processing method. Then, snore events $S_{E}$ and cycle intensity (CI) features proposed by Dafna et al. [15,22] are extracted for use in a context-awareness technique.

### 2.3. Step 2: Random Forest Classification and Final Decision Preparation

#### 2.3.1. Wake/Sleep Random Forest Classification

Random forest classification and final decision preparation are the second major steps of the proposed algorithm, in which two sets of binary random forest classifications are used to achieve likelihood ratios and personal-adjusted thresholds. Random forest classification [25] is a machine learning technique that uses an ensemble of multiple decision trees to train from a bootstrap sample of training data. Each node is split using the finest feature among an arbitrarily-chosen subset of features. Random forest classification is widely used in recent medical research due to its satisfactory performance using minimum sets of training data [26].

The first part of this step is wake/sleep random forest classification, in which the following parameters are achieved: wake/sleep random forest classification likelihood ratios, $R_{\mathrm{WS}}$ = [$R_{\mathrm{WS}}$(1), $R_{\mathrm{WS}}$(2), ⋯ and $R_{\mathrm{WS}}$(*n*)]; and personal-adjusted threshold for wake/sleep decisions, $\gamma_{WS}$. As depicted in Figure 2, the first procedure of wake/sleep random forest classification mainly uses 52 previously-extracted radar features. Certain pre-processing methods of extracted features from the first stage must first take place: feature selection and feature normalization. Among 52 radar features, 44 features that were previously chosen and trained based on feature distribution were used for wake/sleep random forest classification. After feature selection, the following feature normalization technique is applied to each *n*-th feature, F(n) with:

(9) $$∥F\left(n\right)∥=\frac{F\left(n\right)-\mu_{n}}{\sigma_{n}}$$

where *n*, $\mu_{n}$ and $\sigma_{n}$ indicate the index, mean and standard deviation of features, respectively. Using normalized radar features, the wake/sleep random forest classification results in the likelihood of two classes, $p_{0}$ and $p_{1}$, which represent the likelihood of wake and sleep, respectively. After achieving binary likelihood outcomes from each of the *i*-th frames, the likelihood smoothing technique is adopted and interpreted as:

(10) $$p_{c}\left(i\right)=\alpha p_{c}\left(i-1\right)+\left(1-\alpha \right)p_{c}\left(i\right),\mathrm{for}\;c=0,\;1$$

where α is the smoothing parameter set to 0.9. The likelihood ratio of two classes is then calculated frame-wise in the buffer for later usage during the wake/sleep personal-adjusted threshold generation procedure and the final major step.

#### 2.3.2. Personal-Adjusted Threshold

All humans have different sleep patterns, and a personal-adjusted threshold algorithm enables sleep stage classification for all sleep disorder patients. Statistical information from PSG manuals indicates that people tend to have different sleep patterns, which hinders radar receiving different signals at different sleep stages [27]. Our algorithm overcomes this issue by incorporating a newly-proposed personal-adjusted threshold technique as follows.

The array of likelihood ratios $R_{\mathrm{WS}}$ or $R_{\mathrm{RNR}}$ achieved from the random forest classification procedure is sorted in ascending order. The likelihood ratio value located at a certain percentile parameter η that was heuristically determined from a grid-search is then located. This selected value is considered a personal-adjusted threshold for comparison with other likelihood ratios to determine its class. This threshold is saved for later use at the final stage. In the meantime, this threshold is also used to compare the likelihood ratios generated by wake/sleep random forest classifications so that each frame is temporarily classified as awake or asleep. The frames in which it is that assumed patients are asleep are used in the NREM/REM random forest classification procedure.

#### 2.3.3. Snore Event-Based Context-Awareness Method

**Figure 4:**
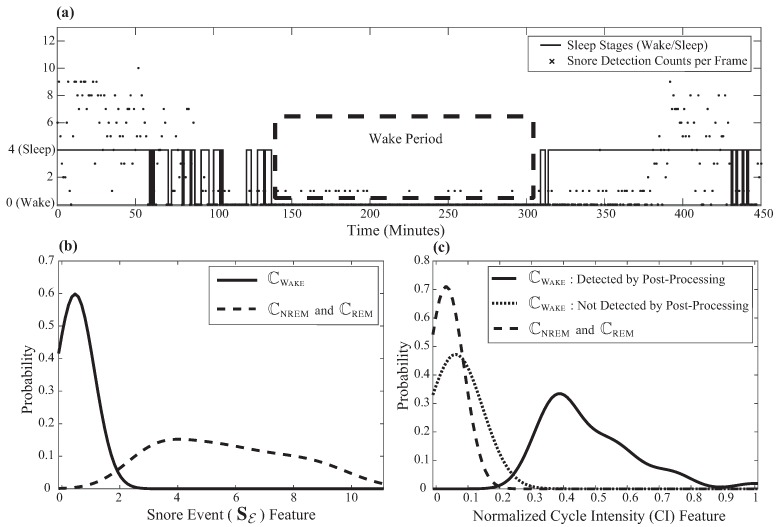
The effect of sound features on wake/sleep classification: (**a**) The detection of snore event features during the wake and sleep class. (**b**) The distribution of snore event feature with respect to the wake and sleep (NREM and REM) class. (**c**) The distribution of the normalized cycle intensity (CI) feature during the wake and sleep class. The solid line describes that the post-processing clearly separates the distribution of the normalized CI feature in the wake class with respect to the sleep class.

The proposed algorithm uses a newly-developed heuristic sound-based context-awareness method that incorporates sound features from snore event detection results that were previously performed in the feature extraction step. Sound features reduce the misclassification error of radar feature-based wake/sleep random forest classification. The concept of this method came from the idea that patients are less likely to snore while awake, as visualized in Figure 4a.

Within the wake period, there are fewer detected snore events than in the sleep period. By analyzing the distribution of $S_{E}$ features with respect to the wake/sleep class, as shown in Figure 4b, the following algorithm is proposed.

#### 2.3.4. NREM/REM Random Forest Classification

From the wake/sleep random forest classification $P_{\mathrm{WS}}$, frames that were classified as sleep are selected for NREM/REM classification. First, the previously-extracted 52 radar features are used to select 35 features that were trained based on feature distribution. This procedure first goes through feature normalization, similar to the wake/sleep classification procedures. The NREM/REM random forest classification procedure is then performed to achieve likelihood ratios $R_{\mathrm{RNR}}$= [$R_{\mathrm{RNR}}$(1), $R_{\mathrm{RNR}}$(2) and ⋯, $R_{\mathrm{RNR}}$(*n*)]. The difference in the NREM/REM classification procedure and wake/sleep classification is that the NREM/REM classification does not use sound features. Moreover, medical knowledge-based percentile parameters were used to generate the NREM/REM personal-adjusted threshold, $\gamma_{\mathrm{RNR}1}$ and $\gamma_{\mathrm{RNR}2}$. Details about this medical knowledge are described in the following section.

### 2.4. Step 3: Post-Processing and Final Sleep Stage Decision

#### 2.4.1. Final Sleep Stage Decision

The final step of our proposed sleep stage classification algorithm concludes about the final sleep stage, $P_{\mathrm{FIN}}$, by determining each 30-s frame as $C_{\mathrm{WAKE}}$, $C_{\mathrm{NREM}}$ and $C_{\mathrm{REM}}$ to indicate the wake class, the NREM class and the REM class, respectively. As shown in Figure 2, the first procedure of this stage is determining the final decision for wake/sleep classification based on the following previously-achieved parameters: array of wake/sleep likelihood ratios, $R_{\mathrm{WS}}$; wake/sleep personal-adjusted threshold, $\gamma_{\mathrm{WS}}$; and snore event detection results, $S_{E}$. Assuming that a person is awake for the first minute of sleep, the wake/sleep decision is performed by comparing the likelihood ratios of each frame with respect to $\gamma_{\mathrm{WS}}$. The algorithm then compensates for misclassification errors by using $S_{E}$, which results in the determination of the final decision for the wake class, $C_{\mathrm{WAKE}}$.

After determining sleep frames, the final NREM/REM classification is performed based on the following previously-achieved parameters: the statistical and medical knowledge-based NREM/REM personal-adjusted threshold, $\gamma_{\mathrm{RNR}1}$; the array of NREM/REM likelihood ratios, $R_{\mathrm{RNR}}$; the NREM/REM personal-adjusted threshold, $\gamma_{\mathrm{RNR}2}$; and the WRMEnAD results. The first step of the NREM/REM final decision is compensating for wake/sleep errors made from the wake/sleep decision by applying WRMEnAD results. The algorithm assumes that a person would not be asleep for 30 s when it was determined that the person was awake during the previous 30 s and the next 30 s. If the person were determined to be asleep from the wake/sleep decision with these conditions, the algorithm modifies the final decision about the sleep stage $P_{\mathrm{FIN}}$(*i*) to the wake class, $C_{\mathrm{WAKE}}$.

**Figure 5:**
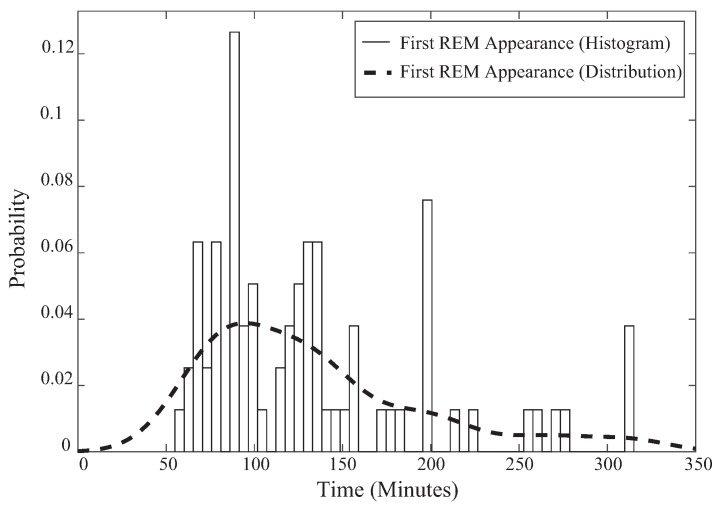
First occurrence of REM.

Next, the likelihood ratios of the sleep determined frames are compared to $\gamma_{\mathrm{RNR}1}$ or $\gamma_{\mathrm{RNR}2}$, which are designed based on the medical knowledge certified by a professor at Hanyang University. $\gamma_{\mathrm{RNR}1}$ is designed based on the fact that sleep stage REM does not appear before the first 50 min of sleep. To confirm this, PSG results of 73 past patients from Hanyang University Hospital that were not included in this experiment were retrieved and examined for the time of first REM occurrence, as shown in Figure 5. Based on this medical (and statistical) knowledge, $\gamma_{\mathrm{RNR}1}$ is generated by weighting more probability of NREM detection compared to REM detection probability before the first 50 min of sleep [28].

#### 2.4.2. Post-Processing 1: Sound-Based Heuristic Wake Period Detection

Based on the common knowledge that it is less likely for a person to snore while awake, a sound-based heuristic wake period detection post-processing technique is used with a previously-extracted normalized CI feature and $S_{E}$. First, eliminate the CI feature during NREM and REM by using the heuristic threshold for $S_{E}$ and the normalized CI feature to avoid misdetection (Figure 4c). If the length of consecutive frames that have the zero CI feature is between 110 and 170 frames, correct the final determined class $P_{\mathrm{FIN}}$(*i*) to the wake class, $C_{\mathrm{WAKE}}$. As shown in Figure 4, the newly-proposed sound-based post-processing technique increases the performance of detecting $C_{\mathrm{WAKE}}$.

#### 2.4.3. Post-Processing 2: Heuristic Knowledge-Based REM Outlier Elimination

To increase the detection of $C_{\mathrm{NREM}}$ and $C_{\mathrm{REM}}$, a heuristic knowledge-based REM outlier elimination technique is used. Although analysis of 73 PSG results statistically confirmed that the REM period sustains less than three frames by 20.9%, this algorithm combines nearby REM predicted frames as one period of REM and then switches the rest of the leftover REM frames that are less than three frames into the NREM class. After this procedure, final post-processing results in the ultimate output of the sleep stage decision.

## 3. Experiments and Results

### 3.1. Experiment

#### 3.1.1. Subjects

We enrolled 33 consecutive patients with suspected OSAwho underwent diagnostic PSG from March to September 2016 (Table 1). We used the Epworth Sleepiness Scale (ESS) for daytime sleepiness and the Pittsburgh Sleep Quality Index (PSQI) for sleep quality. During the sleep study (11 p.m. to 5 a.m.), sound and radar recordings were also performed simultaneously. PSG was conducted and scored manually by a sleep technician under the 2012 ASAMguideline [29]. Nine subjects were excluded due to incomplete recordings of the radar or microphone. We allocated 11 subjects to the training dataset and 13 subjects to the test dataset. There were no statistical differences in the baseline and PSG results between the two groups (Table 1). The study was approved by the Institutional Review Board of Hanyang University Medical Center (HYI-15-135-2).

#### 3.1.2. Polysomnography

Nocturnal PSG (Alice 5; Respironics, Murrysville, PA, USA) was performed in accordance with previous studies and recommendations [29]. Briefly, sleep stages were measured and scored by four-channel electroencephalography, electrooculography and submental electromyography. Respiratory events were diagnosed as apnea or hypopnea, and then, the apnea hypopnea index (AHI) was calculated as the total number of them per hour of sleep. All sleep recordings were scored by following the recent criteria of the second AASMManual for the Scoring of Sleep and Associated Events.

#### 3.1.3. Experimental Environment

**Figure 6:**
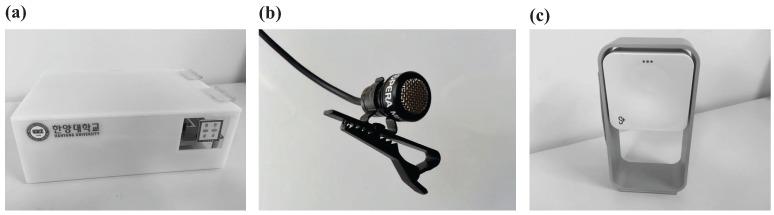
The devices used in experiment: (**a**) RFbeam K-LC5 Doppler radar sensor and ST-200 evaluation system; (**b**) OPERA MP-06 directional microphone sensor; (**c**) ResMed S+.

During the sleep study, we simultaneously collected noncontact data using a K-band 24-GHz Doppler radar transceiver (RFbeam K-LC5, St. Gallen, Switzerland) [17] (Figure 6a), a directional microphone sensor (OPERA MP-06, Seoul, Korea) (Figure 6b) and a commercially-available IoT sleep monitoring device (ResMed S+, San Diego, CA, USA) (Figure 6c) [16]. An additional radar recording device (RFbeam ST-200 Evaluation System, St. Gallen, Switzerland) and an audio recording device (Idam K9-PRO, Seoul, Korea) were placed in the equivalent position.

**Figure 7:**
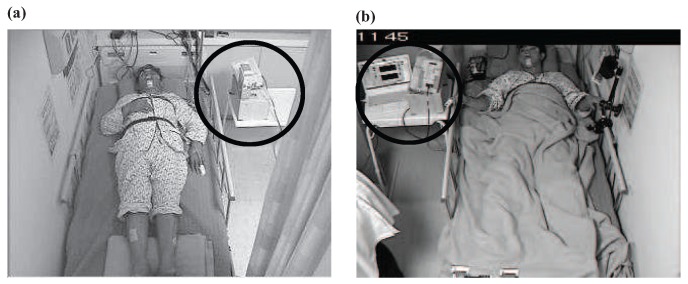
The experimental environment at Hanyang University Hospital: (**a**) Room A; (**b**) Room B.

As shown in Figure 7, the devices were placed at bedside within 50 cm apart from the subject’s side and faced toward the patient’s upper body. All of the data were synchronized with the PSG (Philips Electronics Alice 5 sleepware, Amsterdam, The Netherlands) study [7].

The radar synchronization was performed via the movement signal and the breathing signal appearing in the radar signal, as well as summation of the chest and abdominal respiration channel of PSG, whereas the audio synchronization was performed via the digital audio signal and the snore channel of PSG. Lastly, since ResMed S+ does not provide users with the raw data, the data were time synchronized. The main purpose was to synchronize the PSG channels to all of the data for the manual labeling of sleep stage.

### 3.2. Results

#### 3.2.1. Data Analysis

Despite the relatively small sample size, the subject characteristics are presented as means with standard deviations. Statistical significance (*p* < 0.05) was analyzed using the Mann–Whitney U test. The relationships between snore percentile and the improvement of the algorithm via sensor-fusion were analyzed using Pearson correlation analysis. For diagnostic accuracy of our proposed algorithm and ResMed S+, the accuracy of each sleep stages (wake, NREM and REM) was calculated for every patient. Accuracy was defined as [(true positive cases)/all cases ×100%]. Bland–Altman plot analysis was used to analyze the sleep stage classification performance between the single-sensor and sensor-fusion-based algorithm, as well as between ResMed S+ and the sensor-fusion-based algorithm. All statistical analyses were performed using MATLAB R2016b and Microsoft Excel 2016.

#### 3.2.2. Diagnostic Accuracy of Sleep Stages Tested by Noncontact Devices

**Figure 8:**
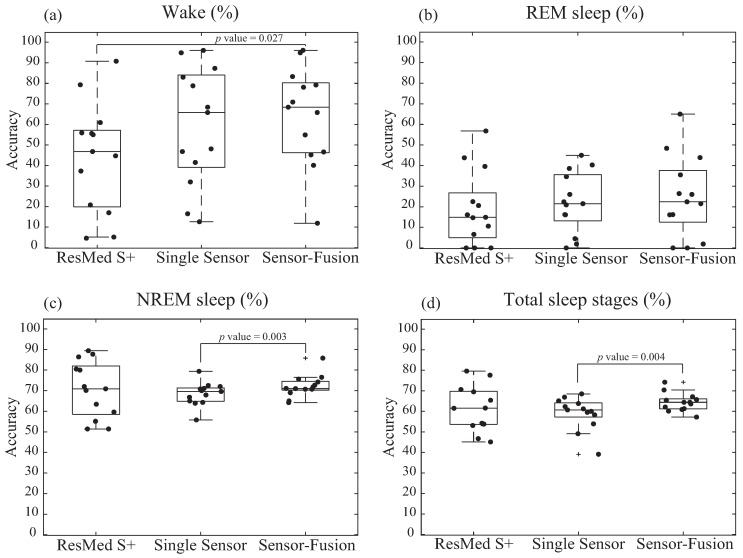
The box plots of: (**a**) wake; (**b**) REM sleep; (**c**) NREM sleep; (**d**) total sleep stage classification of 13 tested subjects. The *x* axis and *y* axis respectively represent the tested sleep monitoring algorithms, and the accuracy (%) with respect to the polysomnographic reference data. The line at the center of the box represents the median, and the upper and lower edges of the box represent the 75th and 25th percentiles. The ends of the whiskers represent 90th and 10th percentiles.

We compared the diagnostic accuracy of sleep stages tested by ResMed S+ and our proposed algorithms (single-sensor and sensor-fusion) in reference to the PSG results (Figure 8 and Table 2).

The sensor-fusion algorithm showed significantly improved accuracy for wake stage than ResMed S+ (43.8% vs. 60.0%, *p* = 0.027). It also showed significantly improved accuracy for NREM sleep (68.4% vs. 71.9%, *p* = 0.003) and total sleep architecture (60.9% vs. 64.4%, *p* = 0.004) than the single-sensor algorithm. However, all three methods of noncontact measurements showed low levels of accuracy for REM sleep detection.

#### 3.2.3. Bland and Altman Analysis of The Results

**Figure 9:**
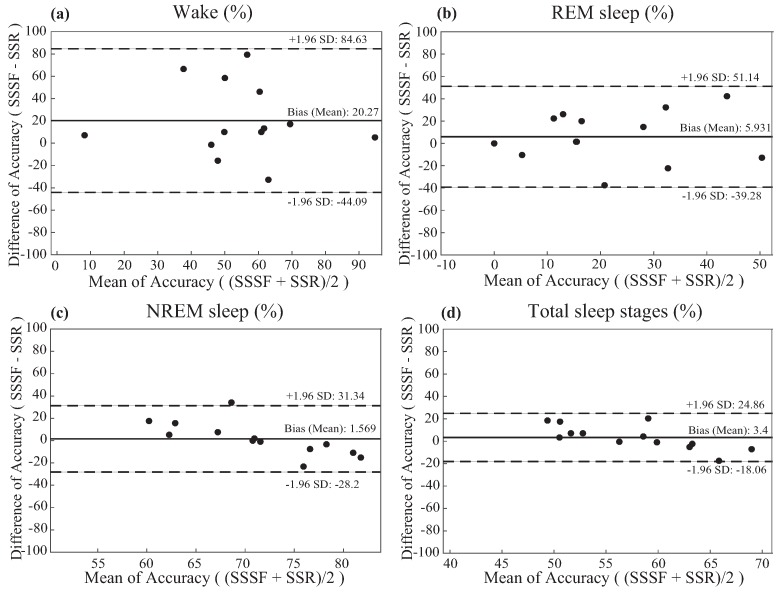
The Bland–Altman plot of: (**a**) wake; (**b**) REM sleep; (**c**) NREM sleep; (**d**) total sleep stage classification accuracy comparing the performance of sleep stages via the sensor-fusion algorithm (SSSF) and the sleep stages via ResMed S+ (SSR). The means of the accuracy of SSSF and SSR are on the *x* axis, and the differences (SSSF-SSR) are on the *y* axis. Indicated in the right are the values, the bias (mean) ± the standard deviation (SD). The bold line represents the mean (bias) difference in the accuracies, and the dotted lines represent the limits of agreement (from −1.96 to +1.96 standard deviations with respect to the mean).

Plotted in Figure 9, the Bland–Altman plots were constructed to analyze the agreement of sleep stage classification measured by ResMed S+ and sensor-fusion. The sensor-fusion and medical knowledge-based algorithm outperformed ResMed S+ on wake detection and REM detection by a total average of 16.2% and 4.5%, respectively (Table 2).

## 4. Discussion and Limitation

### 4.1. Discussion

This study showed that our novel algorithm for sleep stage classification based on the fusion of unobtrusive sensors and medical information allowed us to detect the sleep and wake cycles in a real-life environment with reasonable accuracy for the possibility of commercialization. We propose this as a new approach to sleep stage classification to be embedded in smart home IoT products. PSG has been used as a medical gold standard for evaluating sleep stage, mainly dependent on the EEG [30], electrooculogram (EOG) [31] and electromyogram (EMG) [32] sensors that are attached to the patient. Nonetheless, due to the obtrusiveness of the attachable sensors, the cost and the complexity of the examination, various studies have been sparked in the past. Eliran Dafna et al. classified sleep stages using microphone sensors to detect breathing sounds and to classify if a patient were awake or asleep, but sound data themselves were not enough to determine non-rapid eye movement (NREM) and rapid eye movement (REM) sleep. In a previous study, [11] used a single Doppler radar sensor to determine sleep stage by extracting sleep-related vital signals including breathing rate, heart rate and body motions [12,13,14,33]. However, the experiments were held in an environment in which the reference devices and their manuals were not certified by professional doctors or sleep technologists. Moreover, most previously-proposed sleep stage classification methods were designed based on average users, not patients with sleep disorders who actually require sleep stage information.

By combining the ideas and focusing on the limitations of Tauhidur Rahman et al. [11], Eliran Dafna et al. [15] and ResMed [16], we suggest that fusion of noncontact microphone sensor, K-band 24-GHz Doppler radar sensor data and medical/statistical knowledge-based advanced signal processing algorithms would likely enhance the accuracy of sleep stage classification. Furthermore, using the data for algorithm development, which were certified by a professional sleep technologist and medical doctor at Hanyang University Hospital, provides a trustworthy algorithm that is differentiated from previous studies.

As shown in Figure 8 and Figure 9 and Table 2, studies have shown that the proposed algorithm outperformed a currently commercialized sleep stage classification IoT product, ResMed S+, in classifying sleep stage of 13 actual patients (Table 1) examined at Hanyang University Hospital by a total average of 3.5% (outperformed wake classification by 17.2%, REM by 4.5%). Despite the increased performance of wake and REM detection, the mere 4% difference in total accuracy is because there is less wake and REM sleep epochs than NREM sleep epochs. Therefore, higher accuracy for each sleep stage (wake, REM, NREM) is more crucial than simply achieving higher total accuracy. For this reason, our algorithm is considered to provide higher accuracy of sleep stage classification of the users.

### 4.2. Limitation

One limitation of our study was the trouble of achieving the database due to several factors of the recording device of the K-LC5 sensor, RFBeam ST-200 [17]. First, ST-200 had an extremely unstable software and hardware such that the hardware often becomes disconnected during the overnight examination. Since the PSG examination does not take place on an every-day basis, there was great loss of valuable data. Second, ST-200 is built based on a 16-bit analog-to-digital (ADC) converter such that the resolution is not high enough to support its sensor. Hence, the hardware divided function into LO mode and HI mode, which hindered K-LC5 from the full functioning of its performance. Both modes have the trade-offs of detecting vital signals or limb movements, in which the LO mode was capable of detecting vital signals, whereas HI mode was capable of capturing limb movements. Therefore, we had to choose between LO mode and to develop a novel algorithm to detect limb movements, but the performance of detecting limb movements via LO mode was not as good as HI mode.

There were also more options of high quality radar sensors that could be used for this study [33], but we used a K-band continuous wave Doppler radar due to its low cost. Since our algorithm was developed in order to be commercialized as a low cost smart home IoT product in the future, it was necessary for it to be built affordably, but to be as accurate as possible.

Another limitation was the lack of performance in detecting REM. As shown in Table 2, ResMed S+ and the proposed algorithm both lacked in performance. While REM sleep is usually characterized by rapid eye movements using EOG [31], muscle atonia using EMG [32] and EEG desynchronization, the unobtrusive sensors have difficulties in detecting rapid eye movements due to the body movement artifacts and the sensitivity issues of the sensors. Since REM sleep occurs in every sleep cycle, the REM sleep detection rate may increase if a heuristic approach to analyzing sleep patterns is applied, but it would not be trustworthy. The medically-credible approach can be considered to localize the eyes regardless of the change in posture of the patients, then measuring the rapid eye movement patterns using a highly sensitive radar sensor.

Including the hardware problems indicated above and due to the time-consuming and sparse number of PSG examinations at Hanyang University Hospital, this algorithm was based on a qualifieddatabase of 24 patients during months of research. However, there is potential for developing enhanced algorithm performance once more data are acquired.

## 5. Conclusions

Due to the obtrusiveness, high cost and long procedure of PSG examination to determine sleep stage, there were a number of attempts to develop such algorithms based on unobtrusive sensors in order to enhance the quality of the user’s sleep. However, neither the transparency of the performance was provided, nor was the reference device that was used for algorithm development certified as a gold reference. In this way, a novel approach for classifying sleep stages via low cost and noncontact multi-modal sensor fusion technology of K-band 24-GHz Doppler radar and a microphone sensor is proposed. Our sleep stage classification algorithm was developed based on a gold standard PSG reference-based database and medical knowledge of doctors and sleep technologists at Hanyang University Hospital. This algorithm outscored sleep stage classification accuracy compared to a commercially-available noncontact sleep stage classification IoT product, ResMed S+. Even with a relatively small dataset, the proposed algorithm achieved satisfactory classification performance in real-life scenarios. Ultimately, the choice of commercialization lies in the trade-off between the cost and performance of the sensors. It is crucial that each of the characteristics be confirmed before this algorithm can be officially commercialized as a trustworthy medical IoT product.

## Figures and Tables

**Table 1:** Information of the studied subjects. Data are presented as the mean ± the standard deviation (SD). AHI, apnea hypopnea index.

	Training (11 Patients)	Test (13 Patients)	*p*-Value
**Baseline Information**			
Age (years)	43.45 ± 13.06	40.27 ± 16.3	0.35
Sex (male%)	81.8%	92.3%	0.24
Body Mass Index (BMI) (kg/m${}^{2}$)	24.75 ± 3.76	26.9 ± 4.1	0.11
Medication Status (%)	63.64%	46.15%	0.13
Epworth Sleepiness Scale (ESS)	8.2 ± 3.89	5.54 ± 2.74	0.07
Pittsburgh Sleep Quality Index (PSQI)	8.9 ± 4.37	6.73 ± 3.39	0.13
**PSG Information**			
Total Sleep Time (min)	312.95 ± 38.57	298.05 ± 56.37	0.21
Sleep Efficiency (%)	80.67 ± 6.73	79.71 ± 11.15	0.15
Deep Sleep (%)	0.92 ± 2.63	3.91 ± 6.38	0.09
REM (%)	17.11 ± 5.94	13.76 ± 5.81	0.13
Arousal Index (events/h)	38.85 ± 18.6	41.59 ± 28.61	0.49
Snoring (%)	51.44 ± 32.04	47.51 ± 22.04	0.49
AHI (events/h)	26.75 ± 22.92	44.27 ± 32.95	0.1
RDI(events/h)	30.76 ± 23.48	47.96 ± 32.31	0.11

**Table 2:** Testing accuracy of comparative methods with the proposed sensor-fusion-based algorithm.

Patient #	ResMed S+				Proposed Algorithm							
					Single-Sensor				Sensor-Fusion			
	Wake	REM	NREM	Total Accuracy	Wake	REM	B	Total Accuracy	Wake	REM	NREM	Total Accuracy
P1	20.8%	43.8%	72.0%	**61.6%**	83.0%	21.5%	71.1%	61.2%	79.2%	21.5%	71.1%	60.9%
P2	4.5%	22.5%	80.0%	53.9%	32.0%	45.0%	65.0%	53.9%	70.9%	65.0%	76.5%	**74.2%**
P3	55.6%	14.7%	51.4%	45.1%	41.5%	16.1%	79.4%	60.0%	40.1%	16.1%	85.8%	**63.6%**
P4	37.3%	56.8%	89.4%	**77.6%**	87.3%	38.6%	72.0%	68.5%	83.3%	43.9%	74.2%	70.4%
P5	17.0%	39.6%	87.7%	**79.6%**	96.2%	1.9%	64.3%	62.3%	96.2%	1.9%	64.2%	62.1%
P6	46.8%	20.6%	63.4%	54.2%	46.8%	34.6%	70.0%	60.7%	45.2%	35.5%	71.0%	**61.3%**
P7	4.6%	16.1%	86.4%	**70.6%**	16.5%	40.3%	66.8%	58.3%	11.9%	48.4%	75.6%	65.4%
P8	55.0%	14.9%	70.1%	61.5%	68.4%	16.2%	70.8%	65.1%	68.4%	16.2%	71.8%	**65.7%**
P9	79.3%	6.6%	51.4%	53.8%	48.1%	20.9%	67.9%	49.1%	46.6%	26.4%	69.0%	**57.2%**
P10	55.9%	0.0%	80.5%	**69.5%**	65.8%	22.4%	72.5%	66.9%	65.8%	22.4%	72.7%	67.1%
P11	44.7%	0.0%	59.6%	53.1%	12.6%	0.0%	55.8%	39.1%	54.9%	0.0%	65.0%	**60.1%**
P12	92.1%	10.6%	70.9%	**65.4%**	100.0%	4.5%	63.9%	59.5%	97.4%	0.0%	70.7%	64.4%
P13	60.9%	0.0%	55.1%	46.7%	78.8%	26.0%	69.6%	63.8%	78.1%	26.0%	70.7%	**64.4%**
Total Average	43.8%	21.5%	71.9%	60.9%	59.8%	22.2%	68.4%	59.2%	60.0%	26.0%	71.9%	**64.4%**
